# Prognostic Factors in Dedifferentiated Chondrosarcoma: A Retrospective Analysis of a Large Series Treated at a Single Institution

**DOI:** 10.1155/2019/9069272

**Published:** 2019-12-13

**Authors:** Ruoyu Miao, Edwin Choy, Kevin A. Raskin, Joseph H. Schwab, Gunnlaugur Petur Nielsen, Vikram Deshpande, Ivan Chebib, Thomas F. DeLaney, Francis J. Hornicek, Gregory M. Cote, Yen-Lin E. Chen

**Affiliations:** ^1^Department of Radiation Oncology, Massachusetts General Hospital, Boston, MA 02114, USA; ^2^Department of Medical Oncology, Massachusetts General Hospital, Boston, MA 02114, USA; ^3^Harvard Medical School, Boston, MA 02115, USA; ^4^Department of Orthopedic Oncology, Massachusetts General Hospital, Boston, MA 02114, USA; ^5^Department of Pathology, Massachusetts General Hospital, Boston, MA 02114, USA; ^6^Department of Orthopedic Surgery, University of California Los Angeles, Santa Monica, CA 90404, USA

## Abstract

**Background:**

Dedifferentiated chondrosarcomas (DDCSs) are highly malignant tumors with a dismal prognosis and present a significant challenge in clinical management.

**Methods:**

In an IRB approved retrospective protocol, we identified 72 patients with DDCS treated at our institution between 1993 and 2017 and reviewed clinicopathological characteristics, treatment modalities, and outcomes to analyze prognostic factors.

**Results:**

Femur (44.4%), pelvis (22.2%), and humerus (12.5%) were most commonly involved sites. Twenty-three patients (31.9%) presented with distant metastasis, and 3 (4.2%) of them also had regional lymph node involvement. The median overall survival (OS) was 13.9 months. On multivariate analysis, pathological fracture, larger tumor size, lymph node involvement, metastasis at diagnosis, extraosseous extension, and undifferentiated pleomorphic sarcoma component correlated with worse OS, whereas surgical resection and chemotherapy were associated with improved OS. For progression-free survival (PFS), pathological fracture and metastasis at diagnosis showed increased risk, while chemotherapy was associated with decreased risk. Among patients who received chemotherapy, doxorubicin and cisplatin were significantly associated with improved PFS but not OS. Among patients without metastasis at diagnosis, 17 (34.7%) developed local recurrence. Thirty-one (63.3%) developed distant metastases at a median interval of 18.1 months. On multivariate analysis, R1/R2 resection was related with local recurrence, while macroscopic dedifferentiated component was associated with distant metastasis.

**Conclusions:**

The prognosis of DDCS is poor. Complete resection remains a significant prognostic factor for local control. Chemotherapy with doxorubicin and cisplatin seems to have better PFS. More prognostic, multicenter trials are warranted to further explore the effectiveness of chemotherapy in selected DDCS patients.

## 1. Introduction

Dedifferentiated chondrosarcoma (DDCS) is a type of cartilaginous tumor that is comprised of two distinct components: (1) low-grade chondrogenic components and (2) high-grade noncartilaginous sarcoma. It constitutes 1-2% of all primary bone tumors [1]. Approximately 7–20% of low-grade chondrosarcomas can be expected to dedifferentiate [1–5]. DDCS is slightly more frequent in males. Patients with DDCS are older than those with conventional lesions, with a mean age of around 60 years (range: 15–90 years) [1, 3, 6–9]. The most common sites were the femur and pelvis, followed by humerus and scapula [1, 3, 6].

It has been postulated that the dedifferentiated and cartilaginous components arise from a common primitive mesenchymal progenitor cell with the ability to express features of more than one line of mesenchymal differentiation [10]. However, the separation of the two clones is considered a relatively early event in the tumorigenesis of DDCS and further alterations may lead to the “switch” to a high-grade dedifferentiated chondrosarcoma [11–14]. Histological features of the anaplastic, noncartilaginous component are usually undifferentiated pleomorphic sarcoma (UPS); however, other types of sarcomas include osteosarcomas, fibrosarcomas, angiosarcomas, rhabdomyosarcomas, or leiomyosarcomas [1, 15]. UPS dedifferentiation was reported to be related with poorer outcomes [3]; however, other studies did not reveal any difference in the clinical outcome with different types of the dedifferentiated component [6, 16].

The prognosis of DDCS is dismal. Distant metastasis, especially to the lungs, is common both at the initial presentation and during or relatively soon after initial treatment [6, 17, 18]. The 5-year survival rate can be as low as 7%–24%, with median survival ranging from 5 to 13 months [3, 6, 7, 18–20]. Because of the rarity of DDCS and the insufficient number of large-scale studies, current reports regarding the potential prognostic factors are still inconclusive. One population-based study using the Surveillance, Epidemiology, and End Results (SEER) database suggested that chest wall location predicted better prognosis, whereas larger tumor size, presence of metastases at diagnosis, and no surgical resection were significant predictors of mortality [7]. Another study which utilized the network of member centers of the European Musculo-Skeletal Oncology Society (EMSOS) indicated that pathological fracture, pelvic location, and increasing age predicted poor survival and inadequate excision margins were related to local recurrence and mortality [6]. In addition, there is a lack of convincing evidence on the effectiveness of chemotherapy [1, 3, 6, 18, 20].

In this study, we aim to characterize the impact of clinicopathological features and treatment modalities on the clinical outcomes of patients with DDCS treated at our institution and to investigate prognostic factors.

## 2. Materials and Methods

### 2.1. Patient Selection

Our IRB approved institutional sarcoma database includes 13,412 patients from the 1960s to 2017. Study data were collected and managed using Research Electronic Data Capture (REDCap) electronic data capture tools [21]. The database retrospectively collects data from our institution's clinical records, on patient demographics, primary and secondary tumor characteristics, treatment, follow up, and survival data. We queried the database for patients who were diagnosed with DDCS that was confirmed by pathology review at our institution by expert sarcoma pathologists and were treated in our institution between 1993 and 2017.

**Figure 1:**
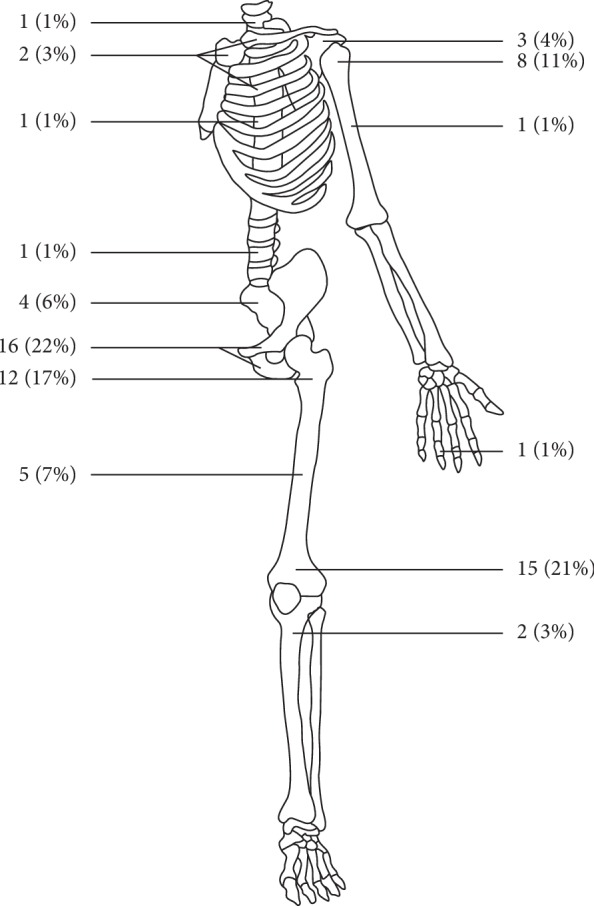
Anatomic sites of dedifferentiated chondrosarcoma.

A total number of 72 patients were identified. Patient demographics and treatment characteristics are summarized in Table 1. Tumor sites are shown in Figure 1. There were 45 men and 27 women with a median age of 60.5 years (range: 29–92 years). Two patients had Ollier's disease, and one had hereditary multiple exostoses. Seventeen patients had a known history of enchondroma (*n* = 5), low-grade chondrosarcoma (*n* = 5), or a bone lesion without further biopsy (*n* = 7) for over 1 year, with the longest present for 15 years. The size of the tumor at the time of diagnosis was available in 66 patients, which averaged 12.4 cm (median: 10.4 cm, range: 3.0–46.0 cm). Twenty-three patients (31.9%) presented with distant metastasis at the time of diagnosis, including lungs (*n* = 20, 27.8%), bones (pelvic bone, rib, skull, and spine, *n* = 5, 6.9%), soft tissue (buttock, chest wall, groin, and thigh, *n* = 4, 5.6%), liver (*n* = 1, 1.4%), and heart (*n* = 1, 1.4%), and 3 (4.2%) of them also had regional lymph node involvement.

### 2.2. Treatment Modalities

Of the 49 patients who did not have identifiable metastases at the initial presentation (M0), 48 received tumor excision (alone in 32, preoperative radiation therapy (RT) in 6, postoperative RT in 6, and both pre- and postoperative RT in 4) and one had RT alone for the primary tumor. Chemotherapy was administered preoperatively alone in 3 patients, postoperatively alone in 7 patients, both preoperatively and postoperatively in 3 patients, and prior to RT in 1 patient. Regimens used include ifosfamide alone (*n* = 1) and combinations of doxorubicin and cisplatin (AP) alone (*n* = 2) or with ifosfamide (*n* = 1)/ifosfamide and etoposide (IE, *n* = 1), methotrexate with AP (MAP, *n* = 5), methotrexate with IE (*n* = 1), IE alone (*n* = 1), and doxorubicin and ifosfamide (AI, *n* = 1) (1 regimen unknown). One patient who was treated with MAP also received the regimen of doxorubicin, ifosfamide and dacarbazine. Of the 6 patients who received neoadjuvant chemotherapy, one had 80% tumor necrosis after MAP, one had 70% necrosis following AP, and another two both had 30% necrosis after the treatment of MAP or methotrexate with IE.

Of the 23 patients who presented with metastasis (M1), 21 underwent surgical excision of the tumor and/or RT to the primary tumor (15 surgery alone, 2 surgery then postoperative RT, 1 surgery with pre- and postoperative RT, and 3 RT alone). Chemotherapy was given to 11 patients, among whom, 2 received preoperative chemotherapy with MAP or methotrexate alone, 6 had postoperative chemotherapy with doxorubicin alone (*n* = 1), AP (*n* = 1), or MAP alone (*n* = 1) or with ifosfamide (*n* = 1)/IE (*n* = 1) (1 regimen unknown), 1 had preoperative methotrexate and cisplatin and postoperative ifosfamide, 1 received AI after RT, and 1 had palliative chemotherapy alone (regimen unknown). Only two of these patients had an assessment of chemotherapy response following neoadjuvant chemotherapy and surgical excision, and the necrosis was 5% with MAP and 70% after methotrexate and cisplatin, respectively.

### 2.3. Statistical Analysis

Statistical significance between groups was analyzed using the chi-squared test or Fisher's exact test for categorical variables and Student *t* test or Mann–Whitney *U* nonparametric test for continuous variables. The estimated overall survival (OS, defined as the time from diagnosis to death from any cause), progression-free survival (PFS, the time from diagnosis to progression or death), local relapse-free survival (LRFS, the time from diagnosis to the first local relapse after treatment or death from any cause), and metastasis-free survival (MFS, the time from diagnosis to the first metastatic relapse after treatment or death from any cause) were derived using the Kaplan–Meier method and compared by the Mantel-Cox log-rank test. The multivariate Cox proportional hazard model was used to investigate significant prognostic factors. The statistical analyses were performed using IBM SPSS Statistics for Windows, version 23.0 (IBM Corp, Armonk, NY). Kaplan–-Meier survival curves were generated in R (version 3.4.1; http://www.r-project.org), using the “survminer” package and the “ggsurvplot” function. All reported *P* values were two-sided. The level of significance was set at *P* < 0.05.

## 3. Results

**Figure 2:**
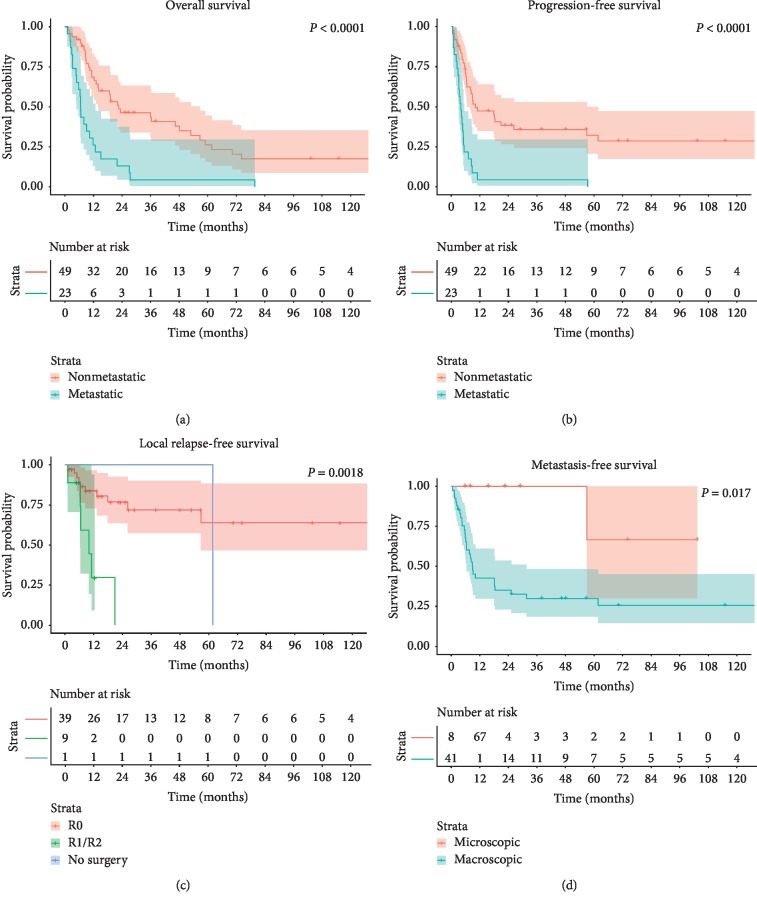
Kaplan–Meier curves. (a) Overall survival and (b) progression-free survival in patients with dedifferentiated chondrosarcoma with and without metastasis at diagnosis. (c) Local relapse-free survival in patients with nonmetastatic dedifferentiated chondrosarcoma according to surgical margin. (d) Metastasis-free survival in patients with nonmetastatic dedifferentiated chondrosarcoma according to the size of dedifferentiation.

The median follow-up was 13 months (range: 1–227 months). The OS rates for all patients were 54.9% (95% confidence interval (CI): 43.1%–66.7%) at 1 year, 35.6% (95% CI: 24.1%–47.2%) at 2 years, and 19.2% (95% CI: 9.0%–29.5%) at 5 years. The median OS for all patients was 13.9 months (95% CI: 6.4–21.5 months). The median OS for patients without metastasis at diagnosis versus those who presented with metastases was 22.6 months (95% CI: 2.0–43.1 months) vs. 6.6 months (95% CI: 6.1–7.1 months) with 68.8% (95% CI: 55.4%–82.1%) vs. 26.1% (95% CI: 7.8%–44.4%) being alive at 1 year and 46.6% (95% CI: 31.9%–61.3%) vs. 13.0% (95% CI: 0%–27.1%) at 2 years following diagnosis, respectively (*P* < 0.001, Figure 2(a)). The 1-, 2-, and 5-year PFS rates in all patients were 33.8% (95% CI: 22.6%–45.0%), 27.7% (95% CI: 16.9%–38.4%), and 21.6% (95% CI: 11.2%–32.1%), respectively.

On univariate analysis, the presence of distant metastasis at diagnosis (*P* < 0.001, Figures 2(a) and 2(b)), pathological fracture (*P* < 0.001), lymph node involvement (*P* < 0.001 for OS, *P*=0.040 for PFS), and positive surgical margin or no surgery (*P*=0.015 for OS, *P*=0.028 for PFS) were associated with poorer OS and PFS, while size of dedifferentiated component (*P*=0.038) also correlated with PFS. There was no difference in OS or PFS based on age, gender, site, tumor size, grade, or RT (Table 2). On multivariate analysis, pathological fracture (hazard ratio (HR): 2.77, 95% CI: 1.53–5.03, *P*=0.001), tumor size larger than 8 cm (HR: 3.43, 95% CI: 1.73–6.79, *P* < 0.001), lymph node involvement (HR: 5.27, 95% CI: 1.26–22.08, *P*=0.023), metastasis at diagnosis (HR: 5.31, 95% CI: 2.63–10.74, *P* < 0.001), extraosseous extension (HR: 5.24, 95% CI: 1.11–24.70, *P*=0.036), and UPS component (HR: 2.44, 95% CI: 1.31–4.57, *P*=0.005) correlated with worse OS, whereas surgical resection (HR: 0.21, 95% CI: 0.08–0.55, *P*=0.002) and chemotherapy (HR: 0.23, 95% CI: 0.12–0.44, *P* < 0.001) were associated with improved OS. Pathological fracture (HR: 2.97, 95% CI: 1.66–5.30, *P* < 0.001), metastasis at diagnosis (HR: 5.21, 95% CI: 2.73–9.95, *P* < 0.001), and chemotherapy (HR: 0.43, 95% CI: 0.24–0.77, *P*=0.005) were also found to be significant prognostic factors for PFS (Table 3).

Among the 25 patients who received chemotherapy, AP was significantly associated with improved PFS (median: 26.3 vs. 6.4 months, *P*=0.007) but not OS. There was no difference in OS or PFS based on osteosarcoma component, UPS, or other regimen received, e.g., methotrexate, MAP, ifosfamide, or IE. Cox multivariate analysis revealed that chemotherapy with AP (HR: 0.29, 95% CI: 0.10–0.80, *P*=0.018) correlated with decreased risk while metastasis at diagnosis (HR: 7.12, 95% CI: 1.18–42.89, *P*=0.032) showed increased risk for PFS.

Among the 49 patients without metastasis at diagnosis, 17 (34.7%) developed a local recurrence. The 1-, 2-, and 5-year LRFS rates were 75.3% (95% CI: 62.3%–88.2%), 66.2% (51.2%–81.3%), and 56.8% (38.8%–74.8%), respectively. Pathological fracture (*P*=0.008), lymphovascular invasion (*P*=0.024), and surgical margin (*P*=0.002, Figure 2(c)) were associated with local recurrence by the univariate analysis. In the Cox model, the HR for R1/R2 resection was 7.51 (95% CI: 2.51–22.46, *P* < 0.001) compared to R0 resection (Table 4). For the 10 patients with either positive margin (*n* = 9) or no surgery (*n* = 1), RT did not result in any difference in LRFS (median: 11.0 months without RT (*n* = 5) vs. 10.2 months with RT (*n* = 5), *P*=0.727). Thirty-one (63.3%) patients later developed distant metastases, including the lungs (*n* = 28, 57.1%), bones (*n* = 7, 14.3%), soft tissue (*n* = 5, 10.2%), mesentery/omentum (*n* = 2, 4.1%), liver (*n* = 1, 2.0%), diaphragm (*n* = 1, 2.0%), adrenal glands (*n* = 1, 2.0%), and appendix (*n* = 1, 2.0%), at a median time of 18.1 months (range: 6.2–30.0 months). The MFS rates were 51.6% (95% CI: 37.1%–66.1%) at 1 year, 44.5% (95% CI: 29.9%–59.2%) at 2 years, and 35.7% (95% CI: 20.8%–50.7%) at 5 years. In the univariate analysis, pathological fracture (*P*=0.017) and size of dedifferentiated component (*P*=0.017, Figure 2(d)) correlated with MFS, whereas only macroscopic dedifferentiated component was found to be a significant factor for MFS (HR: 7.78, 95% CI: 1.06–57.17, *P*=0.044) by the multivariate analysis (Table 4).

## 4. Discussion

The majority of literature investigating prognostic factors affecting DDCS survival were reports on small cohorts from single centers, and only a few more recent ones reported data collected from multiple centers or a large nationwide database (Table 5). Despite significant breakthroughs in cancer therapeutics over the past few decades, the prognosis of this aggressive cancer remains dismal, with a 5-year survival rate ranging between 0% and 29%. Our series had a similar 5-year OS of 19.2% (95% CI: 9.0%–29.5%).

Consistent with what has been reported in the literature (Table 5), DDCS was slightly more frequent in males with a median age of 60.5 years in our cohort. Compared to conventional chondrosarcoma, the older age in DDCS is probably due to the slow indolent process of dedifferentiation, which also makes distant metastasis more likely to be present at diagnosis.

DDCS are associated with a high rate of pathological fractures [4, 6], which increases the risk of local recurrence and predicts poor survival in some studies [6, 25]. In our series, we observed a similar rate of pathological fracture (38.9%) and a negative impact of pathological fracture on both overall survival and disease progression. It is assumed that pathological fracture could lead to local dissemination of tumor cells through hematoma and the difficulty in achieving wide surgical margins in tumor resection [25]. Indeed in our series, R0 resection was achieved in only 54% of all patients with pathologic fractures, compared with 86% in those without fractures (*P*=0.010).

The impact of the histological types of dedifferentiation on the prognosis is still debatable [3, 6, 16]. In the current study, although UPS component did not show significant difference in OS by the univariate analysis, it correlated with a higher risk in mortality by the multivariate analysis. It is possible that certain factors might have more correlation with the UPS component. For example, some patients with UPS component might have been treated more aggressively due to the presumed more aggressive nature, resulting in an improved outcome of UPS in the univariate analysis. Other histological components did not show any influence on OS or PFS.

The value of neoadjuvant or adjuvant chemotherapy remains inconclusive, and the majority of retrospective studies did not reveal any benefit [1, 3, 6, 18, 20]. In the current study, similar to the UPS component, chemotherapy showed a significantly decreased risk in mortality and disease progression by the multivariate analysis but not univariate analysis. Some patients with more aggressive factors might have been treated more aggressively with chemotherapy, which might have masked the effect of chemotherapy in univariate analysis. On the other hand, however, the limited data on the response to neoadjuvant chemotherapy in our series only indicated a relative resistance of DDCS to most classical anti-bone sarcoma drugs. Several studies have indicated that ifosfamide-based adjuvant chemotherapy [8] or doxorubicin monotherapy [22] may offer survival benefit for patients with DDCS. In our series, the combination of doxorubicin and cisplatin was found significantly associated with delayed disease progression. However, the analyses were limited due to the small size of the patients who received chemotherapy and the heterogeneity in chemotherapy regimen during the past three decades.

Surgical resection with complete removal of the tumor is important for LRFS and should be attempted whenever possible. This has also been supported by other studies, although the poor prognosis may influence the radicality of the resection, particularly if it associated with significant morbidity [5, 6, 20]. Consistent with other studies, the prognosis is largely determined by the rapid progress of metastases [1, 7, 20]. There are even fewer data on the influence of RT in the literature. We did not observe any difference in the prognosis by RT, including LRFS, even in patients who did not achieve a complete excision of the tumor.

The limitations of this study cannot be ignored, considering the retrospective nature of this study, long time, and heterogeneity in practice of treatment of our cohort due to the rarity of DDCS. The future of research potentially lies in prognostic, multicenter studies.

## 5. Conclusions

The prognosis of DDCS is poor. Distant metastases are frequently seen either at initial presentation or as later treatment failure. Complete surgical resection remains a significant prognostic factor for local control. Chemotherapy with doxorubicin and cisplatin seems to have better PFS. More prognostic, multicenter clinical trials are warranted to further stratify patients and explore the effectiveness of chemotherapy with both classical anticancer drugs and novel targeted therapies in patients with DDCS.

## Figures and Tables

**Table 1:** The clinicopathological characteristics and treatment modalities in patients with dedifferentiated chondrosarcoma.

	Total	Nonmetastatic	Metastatic
Characteristics	72	49 (68.1%)	23 (31.9%)

Age			
≤60 years	34 (47.2%)	25 (51.0%)	9 (39.1%)
>60 years	38 (52.8%)	24 (49.0%)	14 (60.9%)

Gender			
Female	27 (37.5%)	18 (36.7%)	9 (39.1%)
Male	45 (62.5%)	31 (63.3%)	14 (60.9%)

Pathological fracture	28 (38.9%)	16 (32.7%)	12 (52.2%)

Site			
Extremity	47 (65.3%)	30 (61.2%)	17 (73.9%)
Trunk	25 (34.7%)	19 (38.8%)	6 (26.1%)

Tumor size			
≤8 cm	20 (27.8%)	14 (28.6%)	6 (26.1%)
>8 cm	48 (66.7%)	31 (63.3%)	17 (73.9%)
Discontinuous	4 (5.6%)	4 (8.2%)	0 (0.0%)

Lymph node involvement	3 (4.2%)	0 (0.0%)	3 (13.0%)

Grade			
G2	18 (25.0%)	12 (24.5%)	6 (26.1%)
G3	54 (75.0%)	37 (75.5%)	17 (73.9%)

AJCC 7th edition stage			
II	45 (62.5%)	45 (91.8%)	0 (0.0%)
III	4 (5.6%)	4 (8.2%)	0 (0.0%)
IV	23 (31.9%)	0 (0.0%)	23 (100.0%)

Extra-osseous extension	69 (95.8%)	46 (93.9%)	23 (100.0%)

Lymphovascular invasion			
No	46 (63.9%)	35 (71.4%)	11 (47.8%)
Yes	9 (12.5%)	5 (10.2%)	4 (17.4%)
NA	17 (23.6%)	9 (18.4%)	8 (34.8%)

Dedifferentiated component			
Osteosarcoma	26 (36.1%)	20 (40.8%)	6 (26.1%)
UPS	26 (36.1%)	20 (40.8%)	6 (26.1%)
Fibro/myofibroblastic sarcoma	11 (15.3%)	8 (16.3%)	3 (13.0%)
Undifferentiated spindle cell sarcoma	10 (13.9%)	5 (10.2%)	5 (21.7%)
Rhabdomyosarcoma	2 (2.8%)	1 (2.0%)	1 (4.3%)
Angiosarcoma	1 (1.4%)	1 (2.0%)	0 (0.0%)

Size of dedifferentiated component			
Microscopic	11 (15.3%)	8 (16.3%)	3 (13.0%)
Macroscopic	61 (84.7%)	41 (83.7%)	20 (87.0%)

Chemotherapy	25 (34.7%)	14 (28.6%)	11 (47.8%)

Local treatment			
Surgery	47 (65.3%)	32 (65.3%)	15 (65.2%)
Radiation	4 (5.6%)	1 (2.0%)	3 (13.0%)
Radiation > surgery	6 (8.3%)	6 (12.2%)	0 (0.0%)
Radiation > surgery > radiation	5 (6.9%)	4 (8.2%)	1 (4.3%)
Surgery > radiation	8 (11.1%)	6 (12.2%)	2 (8.7%)
No surgery or radiation	2 (2.8%)	0 (0.0%)	2 (8.7%)

Surgical margin			
R0	53 (73.6%)	39 (79.6%)	14 (60.9%)
R1	2 (2.8%)	2 (4.1%)	0 (0.0%)
R2	11 (15.3%)	7 (14.3%)	4 (17.4%)
No surgery	6 (8.3%)	1 (2.0%)	5 (21.7%)

AJCC, American Joint Committee on Cancer, 7th edition; NA, not available; UPS, undifferentiated pleomorphic sarcoma.

**Table 2:** Prognostic factors for overall survival and progression-free survival in all patients with dedifferentiated chondrosarcoma by univariate analysis.

Variable	Overall survival (months)			Progression-free survival (months)		
	Median	95% CI	*P*	Median	95% CI	*P*
Overall	13.9	6.4–21.5		6.6	3.7–9.4	

Age			0.322			0.424
≤60 years	14.2	5.8–22.5		8.4	6.5–10.4	
>60 years	12.3	1.7–22.9		5.4	3.8–7.0	

Gender			0.445			0.628
Female	11.8	7.8–15.9		5.9	3.6–8.2	
Male	19.0	8.1–29.9		8.0	4.9–11.2	

Pathological fracture			**<0.001**			**<0.001**
No	22.6	0–46.1		9.2	0–21.1	
Yes	7.0	4.6–9.4		4.0	2.3–5.8	

Site			0.304			0.920
Extremity	11.8	7.8–15.9		6.1	4.3–7.9	
Axial	19.0	9.6–28.4		9.0	6.6–11.3	

Tumor size			0.168			0.255
≤8 cm	21.9	15.4–28.4		8.4	4.5–12.3	
>8 cm	11.3	8.1–14.4		6.4	4.6–8.2	
Discontinuous	8.5	0–41.8		1.4	0–108.6	

Lymph node involvement			**<0.001**			**0.040**
No	14.2	6.6–21.7		7.2	4.5–9.8	
Yes	4.7	1.8–7.6		4.0	2.1–5.9	

Distant metastasis			**<0.001**			**<0.001**
No	22.6	2.0–43.1		10.2	0.1–20.2	
Yes	6.6	6.1–7.1		4.0	2.6–5.5	

Grade			0.323			0.077
G2	27.4	0.2–54.6		17.8	0–76.7	
G3	12.3	9.1–15.5		6.4	4.6–8.2	

AJCC stage			**<0.001**			**<0.001**
II	22.6	2.8–42.4		10.2	0–21.4	
III	8.5	0–41.8		1.4	0–108.6	
IV	6.6	6.1–7.1		4.0	2.6–5.5	

Extraosseous extension			0.108			0.073
No	58.9			56.9		
Yes	12.8	8.9–16.6		6.4	3.9–8.9	

Lymphovascular invasion			0.128			0.220
No	14.2	0.8–27.6		8.4	5.2–11.5	
Yes	15.1	0–38.7		5.7	0.8–10.7	
NA	10.3	3.3–17.4		5.1	2.0–8.1	

Osteosarcoma component			0.801			0.669
No	12.3	6.7–17.9		6.6	2.7–10.4	
Yes	19.0	5.2–32.8		6.4	3.6–9.2	

UPS component			0.908			0.596
No	14.2	4.3–24.0		6.1	4.6–7.6	
Yes	12.8	3.4–22.2		8.8	4.1–13.5	

Size of dedifferentiated component			0.172			**0.038**
Microscopic	27.4	0–70.4		56.9	0–147.8	
Macroscopic	12.3	8.5–16.0		6.1	4.8–7.4	

Surgical margin			**0.015**			**0.028**
R0	19.1	8.5–29.8		8.4	5.5–11.3	
R1/R2	12.3	6.1–18.5		6.6	4.2–9.0	
No surgery	6.6	1.5–11.7		3.2	1.7–4.8	

Surgery			0.056			**0.045**
No	6.6	1.5–11.7		3.2	1.7–4.8	
Yes	15.1	8.1–22.0		8.0	5.5–10.6	

Radiation therapy			0.787			0.903
No	13.1	8.7–17.6		7.2	4.6–9.8	
Yes	14.2	0.6–27.7		5.9	1.4–10.4	

Chemotherapy			0.205			0.451
No	10.4	5.1–15.8		5.5	4.1–6.9	
Yes	23.3	5.6–41.1		9.0	6.5–11.4	

AJCC, American Joint Committee on Cancer, 7th edition; CI, confidence interval; NA, not available; UPS, undifferentiated pleomorphic sarcoma.

**Table 3:** Prognostic factors for overall survival and progression-free survival in all patients with dedifferentiated chondrosarcoma by multivariate analysis.

Variable	Overall survival			Progression-free survival		
	HR	95.0% CI	*P*	HR	95.0% CI	*P*
Pathological fracture						
No	1.00			1.00		
Yes	2.77	1.53–5.03	**0.001**	2.97	1.66–5.30	**<0.001**

Tumor size						
≤8 cm	1.00		**0.001**			NS
>8 cm	3.43	1.73–6.79	**<0.001**			
Discontinuous	3.73	0.96–14.56	0.058			

Lymph node involvement						
No	1.00					NS
Yes	5.27	1.26–22.08	**0.023**			

Distant metastasis						
No	1.00			1.00		
Yes	5.31	2.63–10.74	**<0.001**	5.21	2.73–9.95	**<0.001**

Extraosseous extension						
No	1.00					NS
Yes	5.24	1.11–24.70	**0.036**			

UPS component						
No	1.00					NS
Yes	2.44	1.31–4.57	**0.005**			

Surgical resection						
No	1.00					NS
Yes	0.21	0.08–0.55	**0.002**			

Chemotherapy						
No	1.00			1.00		
Yes	0.23	0.12–0.44	**<0.001**	0.43	0.24–0.77	**0.005**

CI, confidence interval; HR, hazard ratio; NS, not significant; UPS, undifferentiated pleomorphic sarcoma.

**Table 4:** Significant prognostic factors for local relapse-free survival and metastasis-free survival in patients with nonmetastatic dedifferentiated chondrosarcoma by univariate and multivariate analyses.

Variable	Univariate			Multivariate		
	Median (months)	95% CI	*P*	HR	95% CI	*P*
Local relapse-free survival	61.7					
Pathological fracture			**0.008**			NS
No	Not reached					
Yes	11.0	0–25.5				
Lymphovascular invasion			**0.024**			NS
No	Not reached					
Yes	11.0	0–22.3				
NA	61.7	0–142.4				
Surgical margin			**0.002**			
R0	Not reached			1.00		**0.004**
R1/R2	10.2	1.2–19.1		7.51	2.51–22.46	**<0.001**
No surgery	61.7			1.98	0.25–15.99	0.520

Metastasis-free survival	18.1	6.2–30.0				
Pathological fracture			**0.017**			NS
No	31.6	0–82.3				
Yes	6.4	3.1–9.7				
Size of dedifferentiated component			**0.017**			**0.044**
Microscopic	Not reached			1.00		
Macroscopic	9.0	6.4–11.6		7.78	1.06–57.17	

CI, confidence interval; HR, hazard ratio; NS, not significant.

**Table 5:** Studies on the survivorship of dedifferentiated chondrosarcoma cohort since 2000.

Authors	Year	No.	Mean age (range)	M : F	5-year OS	Comments
van Maldegem et al. [22]	2019	34	NA	17 : 17	NA	4 centers, unresectable DDCS, positive factor: doxorubicin monotherapy
Nemecek et al. [9]	2018	33	62.2 (22–90)	16 : 17	13.6%	1977–2015, single center, negative factor: CRP
Lex et al. [23]	2018	31	55.6 (33–76)	19 : 12	NA	1995–2016, pelvic DDCS, positive factor: wide surgical margin
Dhinsa et al. [24]	2018	21	64 (35–80)	NA	NA	2000–2010, DDCS with osteosarcoma as predominant component, positive factor: chemotherapy
Strotman et al. [7]	2017	159	65.2 ± 14.7	83 : 76	18%	2001–2011, SEER database positive factor: chest wall tumor, negative factor: size > 8 cm, metastases, no surgical resection
Liu et al. [20]	2017	23	50.4 (32–73)	12 : 11	17.4%	2008–2015, single center, negative factors: axial bone location, lung metastasis, inadequate surgical margin, incorrect diagnosis before surgery, and pathological fractures
Albergo et al. [25]	2015	17	NA	NA	6%	1970–2012, single center, femoral DDCS negative factor: pathological fracture
Kawaguchi et al. [8]	2014	41	58 (26–86)	27 : 14	15%	1986–2010, single center, positive factor: ifosfamide-based adjuvant chemotherapy combined with surgical resection
Italiano et al. [26]	2013	42	NA	NA	NA	1988–2011, 15 centers, advanced DDCS had higher response to chemotherapy than conventional chondrosarcoma
Yokota et al. [18]	2012	9	58.6 (37–86)	4 : 5	0%	1996–2010, single center
Staals et al. [27]	2007	18	46 (22–74)	12 : 6	29%	1970–2002, single center, DDCS that arises in osteochondroma, positive factor: wide surgical resection with adjuvant chemotherapy
Grimer et al. [6]	2007	337	Median 59 (15–89)	179 : 158	24%	1975–2005, 9 centers, negative factors: pathological fracture, pelvic location, increasing age, inadequate margins of excision
Staals et al. [3]	2006	123	59.2 (24–83)	66 : 57	24%	1969–2003, single center, negative factors: metastasis, MFH, high percentage of dedifferentiated component
Bruns et al. [1]	2005	13	59.8 (36–72)	7 : 6	8%	1990–2003, single center, surgery recommended
Dickey et al. [17]	2004	37	66	24 : 18	7%	1986–2000, single center
Mitchell et al. [5]	2000	22	55 (17–81)	15 : 7	18%	1977–1998, single center, positive factors: chemotherapy, wide excision margins

CRP, C-reactive protein; DDCS, dedifferentiated chondrosarcoma; MFH, malignant fibrous histiocytoma; NA, not available; OS, overall survival; SEER, the Surveillance, Epidemiology, and End Results database.

## Data Availability

The data used to support the findings of this study are included within the article.

## References

[1] Bruns J., Fiedler W., Werner M., Delling G. (2005). Dedifferentiated chondrosarcoma?a fatal disease. *Journal of Cancer Research and Clinical Oncology*.

[2] Dahlin D. C., Beabout J. W. (1971). Dedifferentiation of low-grade chondrosarcomas. *Cancer*.

[3] Staals E. L., Bacchini P., Bertoni F. (2006). Dedifferentiated central chondrosarcoma. *Cancer*.

[4] Frassica F. J., Unni K. K., Beabout J. W., Sim F. H. (1986). Dedifferentiated chondrosarcoma. A report of the clinicopathological features and treatment of seventy-eight cases. *The Journal of Bone & Joint Surgery*.

[5] Mitchell A. D., Ayoub K., Mangham D. C., Grimer R. J., Carter S. R., Tillman R. M. (2000). Experience in the treatment of dedifferentiated chondrosarcoma. *The Journal of Bone and Joint Surgery*.

[6] Grimer R. J., Gosheger G., Taminiau A. (2007). Dedifferentiated chondrosarcoma: prognostic factors and outcome from a European group. *European Journal of Cancer*.

[7] Strotman P. K., Reif T. J., Kliethermes S. A., Sandhu J. K., Nystrom L. M. (2017). Dedifferentiated chondrosarcoma: a survival analysis of 159 cases from the SEER database (2001–2011). *Journal of Surgical Oncology*.

[8] Kawaguchi S., Sun T., Lin P. P., Deavers M., Harun N., Lewis V. O. (2014). Does ifosfamide therapy improve survival of patients with dedifferentiated chondrosarcoma?. *Clinical Orthopaedics and Related Research®*.

[9] Nemecek E., Funovics P. T., Hobusch G. M. (2018). C-reactive protein: an independent predictor for dedifferentiated chondrosarcoma. *Journal of Orthopaedic Research®*.

[10] Bridge J. A., DeBoer J., Travis J. (1994). Simultaneous interphase cytogenetic analysis and fluorescence immunophenotyping of dedifferentiated chondrosarcoma. Implications for histopathogenesis. *The American Journal of Pathology*.

[11] Röpke M., Boltze C., Neumann H. W., Roessner A., Schneider-Stock R. (2003). Genetic and epigenetic alterations in tumor progression in a dedifferentiated chondrosarcoma. *Pathology—Research and Practice*.

[12] Yang L., Chen Q., Zhang S. (2009). A novel mutated cell line with characteristics of dedifferentiated chondrosarcoma. *International Journal of Molecular Medicine*.

[13] Simms W. W., Ordóñez N. G., Johnston D., Ayala A. G., Czerniak B. (1995). p53 expression in dedifferentiated chondrosarcoma. *Cancer*.

[14] Röpke M., Boltze C., Meyer B., Neumann H. W., Roessner A., Schneider-Stock R. (2006). Rb-loss is associated with high malignancy in chondrosarcoma. *Oncology Reports*.

[15] Meis J. M. (1991). “Dedifferentiation” in bone and soft-tissue tumors. A histological indicator of tumor progression. *Pathology Annual*.

[16] Dornauer K., Söder S., Inwards C. Y., Bovee J. V. M. G., Aigner T. (2010). Matrix biochemistry and cell biology of dedifferentiated chondrosarcomas. *Pathology International*.

[17] Dickey I. D., Rose P. S., Fuchs B. (2004). Dedifferentiated chondrosarcoma: the role of chemotherapy with updated outcomes. *The Journal of Bone and Joint Surgery-American Volume*.

[18] Yokota K., Sakamoto A., Matsumoto Y. (2012). Clinical outcome for patients with dedifferentiated chondrosarcoma: a report of 9 cases at a single institute. *Journal of Orthopaedic Surgery and Research*.

[19] Capanna R., Bertoni F., Bettelli G. (1988). Dedifferentiated chondrosarcoma. *The Journal of Bone & Joint Surgery*.

[20] Liu C., Xi Y., Li M. (2017). Dedifferentiated chondrosarcoma: radiological features, prognostic factors and survival statistics in 23 patients. *PLoS One*.

[21] Harris P. A., Taylor R., Thielke R., Payne J., Gonzalez N., Conde J. G. (2009). Research electronic data capture (REDCap)—a metadata-driven methodology and workflow process for providing translational research informatics support. *Journal of Biomedical Informatics*.

[22] van Maldegem A., Conley A. P., Rutkowski P. (2019). Outcome of first-line systemic treatment for unresectable conventional, dedifferentiated, mesenchymal, and clear cell chondrosarcoma. *The Oncologist*.

[23] Lex J. R., Evans S., Stevenson J. D. (2018). Dedifferentiated chondrosarcoma of the pelvis: clinical outcomes and current treatment. *Clinical Sarcoma Research*.

[24] Dhinsa B. S., DeLisa M., Pollock R., Flanagan A. M., Whelan J., Gregory J. (2018). Dedifferentiated chondrosarcoma demonstrating osteosarcomatous differentiation. *Oncology Research and Treatment*.

[25] Albergo J. I., Gaston C. L., Jeys L. M. (2015). Management and prognostic significance of pathological fractures through chondrosarcoma of the femur. *International Orthopaedics*.

[26] Italiano A., Mir O., Cioffi A. (2013). Advanced chondrosarcomas: role of chemotherapy and survival. *Annals of Oncology*.

[27] Staals E. L., Bacchini P., Mercuri M., Bertoni A. F. (2007). Dedifferentiated chondrosarcomas arising in preexisting osteochondromas. *The Journal of Bone and Joint Surgery-American Volume*.

